# Design-Considerations regarding Silicon/Graphite and Tin/Graphite Composite Electrodes for Lithium-Ion Batteries

**DOI:** 10.1038/s41598-018-33405-y

**Published:** 2018-10-26

**Authors:** Manuel Otero, Christopher Heim, Ezequiel P. M. Leiva, Norbert Wagner, Andreas Friedrich

**Affiliations:** 1 IFEG, Facultad de Matemáticas, Astronomía y Física, Universidad Nacional de Córdoba, Córdoba, Argentina; 2 INFICQ, Departamento de Química Teórica y Computacional, Facultad de Ciencias Químicas, Universidad Nacional de Córdoba, Córdoba, Argentina; 3 German Aerospace Center (DLR), Institute of Engineering Thermodynamics, Pfaffenwaldring 38-40, 70569 Stuttgart, Germany; 4 Institute for Energy Storage, University of Stuttgart, Pfaffenwaldring 6, 70550 Stuttgart, Germany

## Abstract

An analytical model is proposed to investigate properties of composite electrodes that utilize more than one active material. We demonstrate how the equations can be applied to aid in the design of electrodes by comparing silicon-graphite and tin-graphite composite negative electrodes as examples with practical relevance. Based on simple assumptions, the results show how volume expansion tolerance and initial porosity are important factors for the achievable gravimetric and volumetric capacities as well as volumetric energy density. A Si-alloy/graphite composite electrode is used as an experimental system to corroborate the formulated analysis. Kinetic limitations are also addressed based on a novel heuristic approach.

## Introduction

Due to the recent increase in interest towards battery electric vehicles, hybrid electric vehicles and longer lasting consumer electronic devices, significant effort by industrial and scientific organizations has been put into the development of higher energy density batteries. Generally, the energy density of a lithium-ion battery can be increased by increasing the specific capacity of the used active materials as well as by increasing the operating voltage of the whole cell or a combination of the two. It is commonly accepted that the biggest gains can be achieved by improving or changing the positive electrode materials, since generally commercially utilized cathode materials like lithium cobalt oxide (LCO) have a specific capacity of 140 *mAh/g*^1^, which is rather low compared to the specific capacity of 330 *mAh/g*^2^ for standardly utilized graphite negative electrode material. Nevertheless, changing the negative electrode materials can lead to gains in energy density in commercially used Li-ion batteries. Probably the most investigated candidate materials to replace graphite are silicon (Si) and tin (Sn)^3^. Both materials offer a different chemistry as compared with commonly used intercalation materials like graphite and LCO, where lithium-ions are incorporated into free intercalation-sites inside the existing crystal-lattice. Silicon and tin electrochemically react with lithium-ions to form an alloy. This reaction-mechanism makes it possible to achieve high specific and volumetric capacities. The downside of the alloying reaction is a large volume change *e*_*si*_ of up to 280 vol.-% for the highest lithiated silicon phase at room-temperature, Li_15_Si_4_^4^. Compared with the small volume change e_G_ = 10 vol.-% of graphite upon full lithiation, the volume expansion of alloying active materials is rather substantial.

It is speculated that some manufacturers are already incorporating small amounts of silicon into their graphite negative electrodes to increase energy density^5^. However, the practical application of silicon in combination with graphite in composite electrodes, where two active materials are employed in one electrode structure, is still scarce.

**Figure 1:**
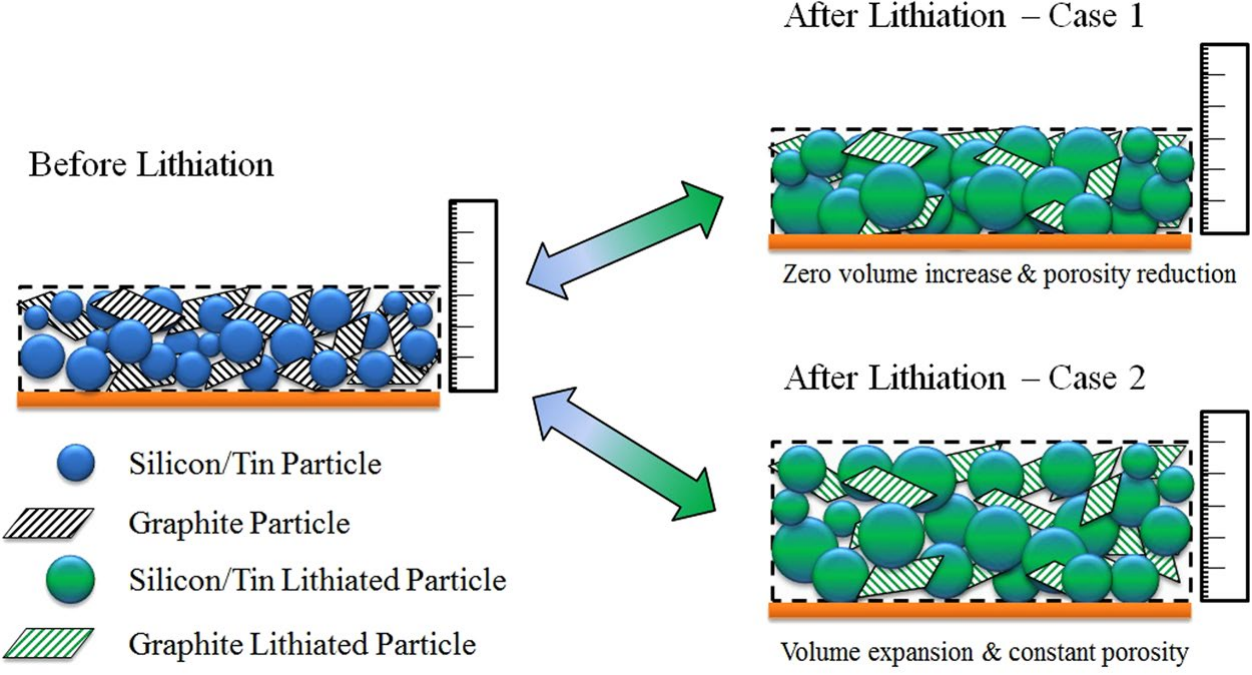
Schematic illustration of the cross-section of a composite electrode. Two possible cases are studied for the electrode after lithiation. Case-study 1 (upper right) considers zero electrode expansion and thus a decrease in porosity, while case-study 2 (lower right) considers a constant porosity and a free electrode expansion. The inactive materials are omitted in the illustration for clarity but will be considered in the calculations.

In the present work, we derive analytical model equations that can be utilized using simple spread-sheet software, and describe the relevant material and electrode parameters needed for making the calculations. These equations utilize a full-electrode expansion factor E, which has similarities to the swelling coefficient used in the model by Gomadam *et al*.^6^. This parameter E describes an expansion tolerance of a battery-full-cell stack and can be used to investigate different cases that are relevant for the conceptual design of electrodes with regards to porosity and thickness increase. Two different cases are defined for the present theoretical investigation of composite electrodes and to derive the theoretically expected gravimetric and volumetric capacities, as well as the anode volumetric energy density as considered in the work of Obrovac *et al*.^7^. For the first case-study, illustrated on the upper right of Fig. 1, the expansion factor will be set to zero, E = 0, which means that the battery stack is not allowed to increase in volume. This case is similar to the calculation performed by Dash *et al*.^8^, but our results lead to different conclusions using the equations given below. In the second case-study considered, illustrated on the bottom right of Fig. 1, the initial porosity *P*_*A*_ and final porosity *P*_*ALi*_ are fixed to the same constant value as experimentally observed by Du *et al*.^9^ and the expansion of the cell upon lithiation is calculated.

To illustrate the applicability of the present approach, we consider experimentally measured capacity values of a silicon alloy/graphite composite electrode and contrast them with the theoretical results based on the equations developed here. Pure material electrode data will be presented, as well as measured data for an electrode composed of 25 wt.-% silicon alloy, 63 wt.-% graphite and 12 wt.-% binder.

Finally, on the basis of experimental data from the literature, we analyze the dependency of the capacity of the material on the delithiation current for different nanostructured silicon electrodes. To the best of our knowledge, we find for the first time a very general behavior that can be used heuristically to study the influence of kinetics properties on the capacity of the material.

## Results and Discussion

### Density, porosity and specific gravimetric and volumetric capacities

The initial density of a porous composite electrode, *ρ*_*A*_, can be simply calculated^8^ as a function of the weight fraction (in wt.-%), *w*_*j*_, and initial densities (in g/cm^3^), *ρ*_*j*_, of the components (silicon, tin, graphite, binder, conductive carbon, etc.) and the initial porosity (in vol.-%) of the unlithiated negative electrode, *P*_*A*_, according to:

1 $${\rho }_{A}=\frac{100-{P}_{A}}{{\sum }_{j}\frac{{w}_{j}}{{\rho }_{j}}}$$

The quality of electrodes can be studied as a function of the gravimetric capacity, *G*_*A*_(in mAh/g)^10^, which for composite materials is defined as^11^:

2 $${G}_{A}=\frac{{\rm{A}}{\rm{m}}{\rm{o}}{\rm{u}}{\rm{n}}{\rm{t}}\,{\rm{o}}{\rm{f}}\,{\rm{c}}{\rm{h}}{\rm{a}}{\rm{r}}{\rm{g}}{\rm{e}}\,({\rm{l}}{\rm{i}}{\rm{t}}{\rm{h}}{\rm{i}}{\rm{u}}{\rm{m}})}{{\rm{M}}{\rm{a}}{\rm{s}}{\rm{s}}\,{\rm{o}}{\rm{f}}\,{\rm{t}}{\rm{h}}{\rm{e}}\,{\rm{u}}{\rm{n}}{\rm{l}}{\rm{i}}{\rm{t}}{\rm{h}}{\rm{i}}{\rm{a}}{\rm{t}}{\rm{e}}{\rm{d}}\,{\rm{a}}{\rm{n}}{\rm{o}}{\rm{d}}{\rm{e}}}={\sum }_{j}\frac{{w}_{j}\cdot {s}_{j}}{100}$$

where *s*_*j*_ represents the gravimetric capacity (in mAh/g) of each of the components. Another important quantity that may be used to characterize electrodes is the volumetric capacity, *V*_*A*_ (in mAh/cm^3^). Especially in automotive applications, in many cases restrictions in space are more severe than weight restrictions, which makes the volumetric capacity increasingly relevant compared to the gravimetric capacity. *V*_*A*_ can be related to the gravimetric capacity by the following equation

3 $${V}_{A}=\frac{{\rm{A}}{\rm{m}}{\rm{o}}{\rm{u}}{\rm{n}}{\rm{t}}\,{\rm{o}}{\rm{f}}\,{\rm{c}}{\rm{h}}{\rm{a}}{\rm{r}}{\rm{g}}{\rm{e}}\,({\rm{l}}{\rm{i}}{\rm{t}}{\rm{h}}{\rm{i}}{\rm{u}}{\rm{m}})}{{\rm{V}}{\rm{o}}{\rm{l}}{\rm{u}}{\rm{m}}{\rm{e}}\,{\rm{o}}{\rm{f}}\,{\rm{t}}{\rm{h}}{\rm{e}}\,{\rm{a}}{\rm{n}}{\rm{o}}{\rm{d}}{\rm{e}}}={G}_{A}\cdot \rho $$

where *ρ* is the density of the electrode. Alloying materials like Si and Sn considerably expand upon lithium uptake, so that the volume and density of the electrode changes upon lithiation^12^, and so will the volumetric capacity. A realistic analysis should consider the density of the electrode at its full volume expansion^7^, since the battery will need to have space for holding both charged and discharged states. The density at full lithiation *ρ*_*ALi*_ can be calculated in a similar way to *ρ*_*A*_:

4 $${\rho }_{ALi}=\frac{100-{P}_{ALi}}{{\sum }_{j}\frac{{w}_{j}}{{\rho }_{j}}+{\sum }_{j}\frac{{w}_{j}}{{\rho }_{j}}\frac{{e}_{j}}{100}}$$

where ${e}_{j}=100\cdot ({V}_{j}^{Lithiated}/{V}_{j}^{Unlithiated}-1)$ represents the volumetric expansion (in vol.-%) of each material upon lithiation and *ρ*_*ALi*_ is the anode porosity at full lithiation (in vol.-%). Using the lithiated electrode density, *ρ*_*ALi*_, the volumetric capacity can be calculated as:

5 $${V}_{A}={G}_{A}\cdot {\rho }_{ALi}={\sum }_{j}\frac{{w}_{j}\cdot {s}_{j}}{100}\cdot \frac{100-{P}_{ALi}}{{\sum }_{j}\frac{{w}_{j}}{{\rho }_{j}}+{\sum }_{j}\frac{{w}_{j}}{{\rho }_{j}}\frac{{e}_{j}}{100}}$$

Now we address the calculation to find a relation between the initial porosity *P*_*A*_ and the final porosity *P*_*ALi*_. A relation between these two quantities can be obtained by studying the difference between initial (unlithiated) volume, *V*^*i*^, and final (lithiated) volume, *V*^*f*^:

6 $${\rm{\Delta }}V={V}^{f}-{V}^{i}=(\frac{{P}_{ALi}\cdot {V}^{f}}{100}+{\sum }_{j}{V}_{j}\cdot (1+\frac{{e}_{j}}{100}))-(\frac{{P}_{A}\cdot {V}^{i}}{100}+{\sum }_{j}{V}_{j})$$

where *V*_*f*_ denotes the initial volume occupied by the component *j* of the composite electrode. Defining the initial volumetric fraction (in vol.-%) of each material as ${v}_{j}=100\cdot {V}_{j}/{V}^{i}$ and solving for the porosities yields:

7 $${P}_{A}={\sum }_{j}\frac{{v}_{j}\cdot {e}_{j}}{100}+{P}_{ALi}\frac{{V}^{f}}{{V}^{i}}+100\cdot (1-\frac{{V}^{f}}{{V}^{i}})$$

which is the relationship we sought between the initial and final porosities.

### Case-Study 1: Zero expansion of the electrode

If the battery structural disposition does not allow for an expansion of the electrode^8^, then the total volume is fixed (*V*^*i*^ = *V*^*f*^). As a consequence the volume expansion of active materials due to lithiation will decrease the available porosity. Therefore considering a suitable initial porosity to accommodate the volume changes is mandatory. Thus required initial porosity will be given by:

8 $${P}_{A}={\sum }_{j}\frac{{v}_{j}\cdot {e}_{j}}{100}+{P}_{ALi}$$

Compared to a similar analysis performed by Dash *et al*.^8^ our derivation of the relevant equation for the initial porosity depends on the volume fractions of the constituent materials *v*_*si*_ and *v*_*G*_ (a detailed comparison to the mentioned ref.^8^ and an alternative derivation is given in the Supplementary Information). Using the relation between initial densities, volume fractions and weight fractions we calculate the volume fraction of component j to be:

9 $${v}_{j}=\frac{{\rho }_{A}}{{\rho }_{j}}{w}_{j}$$

One obtains the following relation that states the required initial porosity to have no increase in volume upon full lithiation, reaching a final porosity *P*_*ALi*_.

10 $${P}_{A}=\frac{{P}_{ALi}{\sum }_{j}\frac{{w}_{j}}{{\rho }_{j}}+{\sum }_{j}\frac{{w}_{j}{e}_{j}}{{\rho }_{j}}}{{\sum }_{j}\frac{{w}_{j}}{{\rho }_{j}}+{\sum }_{j}\frac{{w}_{j}}{{\rho }_{j}}\frac{{e}_{j}}{100}}$$

The highest volumetric capacities will be obtained when the expansion, upon lithiation, of the active materials will completely fill the initial pores of the electrode, which gives the porosity at full lithiation *P*_*ALi*_ = 0. Zero porosity or low porosity will negatively affect the transport properties which are not included in the presented analysis^13^. These dynamic properties could be investigated by numerical simulation^14^ of a porous electrode or by cycling experiments in combination with BET or mercury intrusion measurements for example and will be a topic of future work endeavors.

**Figure 2:**
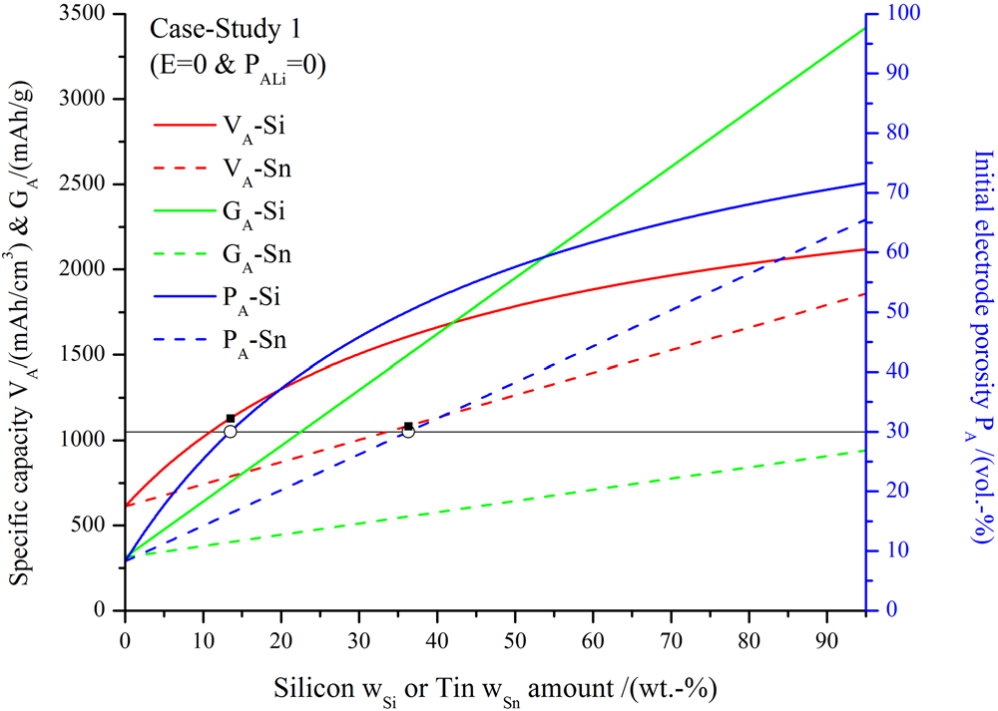
Volumetric capacities (*V*_*A*_ in mAh/cm^3^) (red), gravimetric capacities (*G*_*A*_ in mAh/g) (green) and required initial porosity (*P*_*A*_ in vol.-%) (blue) of silicon/graphite electrodes (full lines) and tin/graphite electrodes (broken lines). The ■ (black squares) indicate the *V*_*A*_ at *P*_*A*_ = 30 vol.-% for each type of electrode. These values were obtained as follows: First, the intersections between the blue curves and the black line marking 30 vol.-% of *P*_*A*_ were found; this yielded the value of *w*_*si*_ or *w*_*sn*_ corresponding to this porosity, marked with ○ (empty circles) in the Figure. Then, for this composition, the volumetric capacity *V*_*A*_ was found from the intersection of a vertical line corresponding to this value and the red lines. The parameters considered were *V*^*i*^ = *V*^*f*^, *P*_*ALi*_ = 0, ${\rho }_{Si}=2.3\frac{g}{c{m}^{3}}$, ${\rho }_{Sn}=7.36\frac{g}{c{m}^{3}}$, ${\rho }_{G}=2.24\frac{g}{c{m}^{3}}$, ${\rho }_{IM}=1.1\frac{g}{c{m}^{3}}$^8^, ${s}_{Si}=3600\frac{mAh}{g}$, ${s}_{Sn}=990\frac{mAh}{g}$, ${s}_{G}=330\frac{mAh}{g}$, *e*_*si*_ = 280 vol.-%, *e*_*si*_ = 257 vol.-%^12^, *e*_*G*_ = 10 vol.-%, *w*_*IM*_ = 5 wt.-%.

In Fig. 2 we show the results for specific volumetric and gravimetric capacity and required initial porosity for case-study 1 (*V*^*i*^ = *V*^*f*^ and *P*_*ALi*_ = 0). Negative electrodes composed of silicon/graphite (full lines) and tin/graphite (broken lines) are considered, varying the weight fractions *w*_*si*_ and *w*_*sn*_ respectively, maintaining a fixed amount of inactive materials *w*_*IM*_ = 5 wt.-% and varying the graphite composition in accordance.

Several interesting conclusions can be obtained from Fig. 2. The gravimetric capacity *G*_*A*_ (green) of Si and Sn composite negative electrodes increases linearly with the weight fraction as expected, where Si has a 264% higher gravimetric capacity for a 95 wt.-% content with respect to Sn. Compared to a recent publication^8^, the volumetric capacity (red) of the composite electrodes increases monotonically as a function of weight fraction of Si or Sn and does not show a threshold value for the useful amount of Si in the electrode. A threshold value for the amount of Si inside a composite electrode is unlikely and a discussion based on the derived equations can be found in the Supplementary Information. However it is interesting to notice the functional difference of *V*_*A*_ as a function of the weight-fraction of Si and Sn inside the electrode-coating. For Sn the increase is roughly linear over the whole range, but for Si there is a rapid increase in the range of low Si content and a decreasing impact for an increasing amount of Si content. This functional difference in the curves has the following consequences: for a high metal content, say 95 wt.-%, the volumetric capacity of Si surpasses that of Sn only by 14%. On the other hand, for a moderate metal content, say 30 wt.-%, the difference between volumetric capacities is more pronounced, in this case in favor of Si with a 50% increased *V*_*A*_. The initial porosity *P*_*A*_ required to allow the expansion of the active materials upon lithiation without total electrode volume expansion (*E* = 0) has a different functional behavior for Si and Sn, reflecting the behavior of *V*_*A*_. In this regard it is important to point out that by increasing silicon content in the composite, in the region of low weight-fractions, will require pronounced increases in initial porosity *P*_*A*_. This quickly leads to initial porosity values that might not have any practical relevance. It is interesting to notice that if one fixes the working porosity of the prepared electrode to 30%, which is a typical value for commercially produced electrodes^9^, the volumetric capacity *V*_*A*_ of both materials is approximately equal (only a 4% difference in favor of Si as shown by the marked black squares) while the specific capacity *G*_*A*_ results in a 36% difference in favor of Si. This is because, although Si has a higher specific capacity, a higher weight fraction of Sn is possible for the same fixed anode porosity *P*_*A*_ value. From a practical point of view this analysis shows that only adding a second active material to a graphite electrode based on a higher specific capacity, which is oftentimes the “performance indicator” used in publications of new materials, is not enough to assess the impact of blending two materials in an electrode. Since commercially used graphite electrodes have a low volume expansion and a low potential versus lithium, the addition of a second active material needs to be well considered. The case study presents an approach to acquire a more comprehensive idea of the effects caused by the addition of a second active material whilst requiring commonly measured data. This can guide further experimental efforts if for example expansion tolerances for cells inside battery-packs are known.

The actual amount of inactive material employed depends on the type of active materials used and the optimization done in the fabrication of the electrode. While research publications use relatively high amounts of inactive material, this is minimized as much as possible in practical applications. Calculations with different amounts of inactive material are included in Supplementary Information.

### Case-Study 2: Constant porosity and free electrode expansion

A second interesting case for a theoretical investigation is the assumption that the volume expansion causes an increase in electrode thickness while the porosity stays constant, as experimentally observed^9^. For this case volumetric capacity is obtained by equation (5) considering *P*_*ALi*_ = *P*_*A*_ and the electrode is allowed to expand freely. This electrode volume increase is studied by incorporating the electrode expansion factor $E=100\cdot ({V}^{f}/{V}^{i}-1)$ into the equations. Using the density definition of the unlithiated (see eq. (1)) and lithiated (eq. (4)) negative electrode; the expansion can be calculated as:

11 $$E=\frac{{\sum }_{j}\frac{{w}_{j}}{{\rho }_{j}}{e}_{j}}{{\sum }_{j}\frac{{w}_{j}}{{\rho }_{j}}}$$

**Figure 3:**
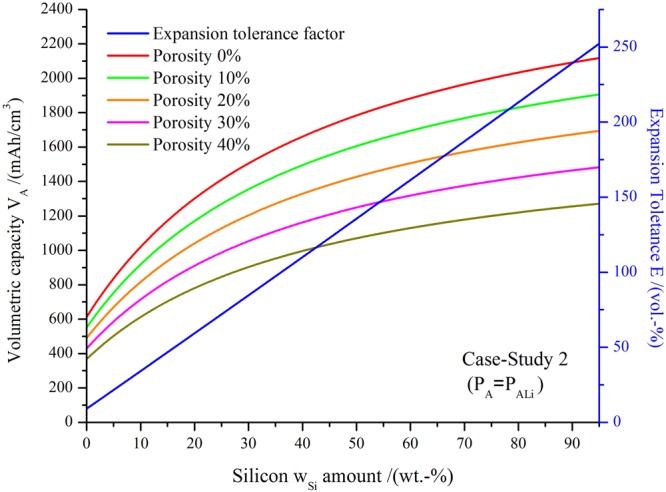
Volumetric capacities (*V*_*A*_ in mAh/cm^3^) of silicon/graphite composite electrodes for different porosities (0, 10, 20, 30 and 40 vol.-%) under the condition of a constant porosity are shown. The expansion tolerance E (in vol.-%) required to keep a constant porosity upon lithiation is drawn with the blue line. The parameters considered were, *P*_*ALi*_ = *P*_*A*_, ${\rho }_{Si}=2.3\frac{g}{c{m}^{3}}$, ${\rho }_{G}=2.24\frac{g}{c{m}^{3}}$, ${\rho }_{IM}=1.1\frac{g}{c{m}^{3}}$^8^, ${s}_{Si}=3600\frac{mAh}{g}$, ${s}_{G}=330\frac{mAh}{g}$, *e*_*si*_ = 280 vol.-%, *e*_*G*_ = 10 vol.-%, *w*_*IM*_ = 5 wt.-%.

Figure 3 shows the volumetric capacity for negative electrodes of different porosities in the whole range of compositions of silicon/graphite, maintaining a fixed amount of inactive materials *w*_*IM*_ = 5 wt.-%.

For case-study 2 and for a given Si amount, the volumetric capacity decreases for increasing percentage of porosity as expected while maintaining a similar functional form. Comparable behavior is observed for tin/graphite negative electrodes (see Supplementary Information). The expansion tolerance E required for the negative electrode material is the same in all cases and the increase is roughly linear with the amount of silicon added (blue line).

### Average potentials and volumetric energy density

To compare different battery-types, the anode volumetric energy density, *ED*_*Vol*_ (in Wh/L), is analyzed. *ED*_*Vol*_ can be calculated as a function of the average discharge potential of the anode, ${U}_{-}^{Average}$, the volumetric capacity of the anode, *V*_*A*_ and the average discharge potential of a cathode, ${U}_{+}^{Average}$, according to^3^:

12 $$E{D}_{Vol}=({U}_{+}^{Average}-{U}_{-}^{Average})\,\cdot \,{V}_{A}$$

where $({U}_{+}^{Average}-{U}_{-}^{Average})$ is the average discharge potential of the cell, *U*_*Cell*_. For the present purposes, we remind that ${U}_{-}^{Average}$ depends on the composition of the negative electrode. If the negative electrode components absorb lithium at different potentials and one considers that each component keeps its characteristics voltage profile, the average potential can be approximated by^15–17^:

13 $${U}_{-}^{Average}=\frac{{\sum }_{j}{U}_{j}^{Average}\cdot {w}_{j}{s}_{j}}{{\sum }_{j}{w}_{j}{s}_{j}}$$

**Figure 4:**
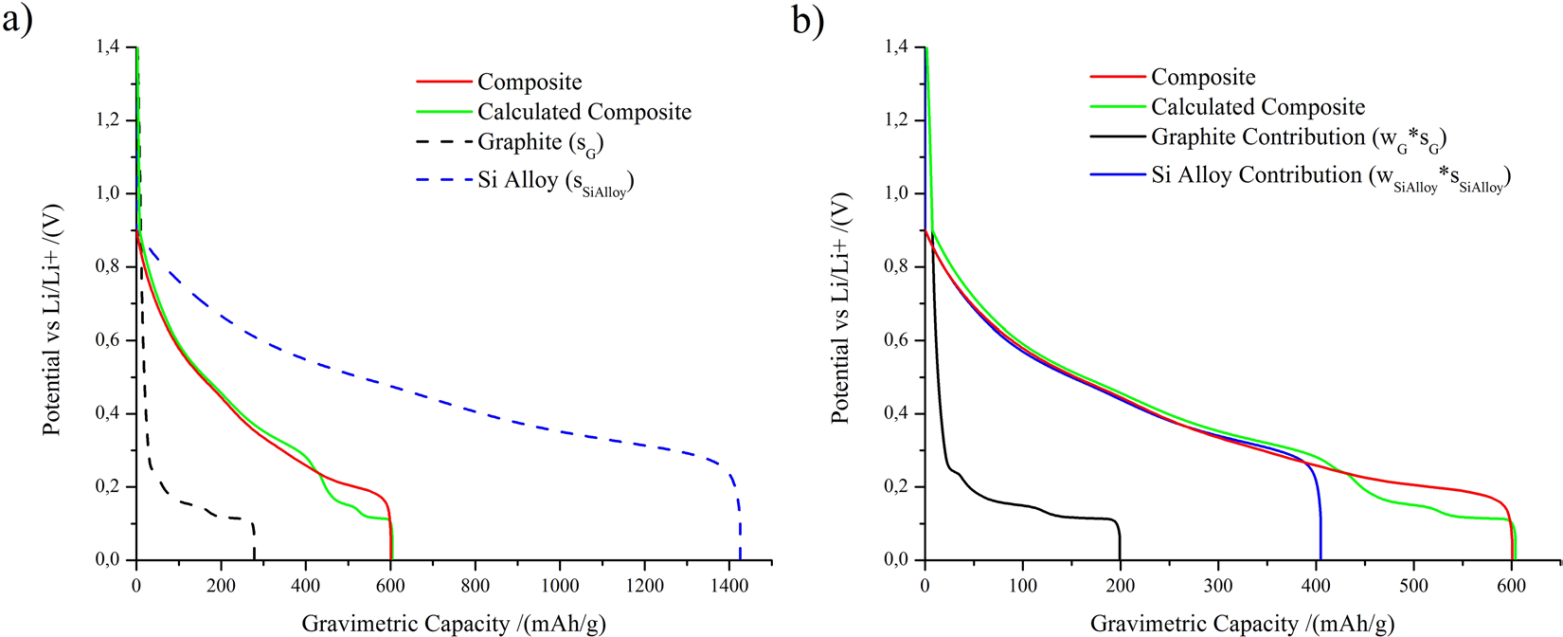
Second delithiation potential-capacity curves for a Si alloy-graphite composite electrode measured (red) and estimated (green), according to equation (13). (**a**) Includes the curves for the Si alloy electrode (dashed blue) and a graphite electrode (dashed black) as measured in half-cells; (**b**) shows the curves for the scaled Si alloy electrode (blue) and a scaled graphite electrode (black) that were used for the calculation of the average potential. Scaling of the contribution is done taking into account the weight fraction of each component in the composite electrode, 25 wt.-% for Si alloy and 63 wt.-% for graphite. The calculated composite curve (green) is obtained adding up the capacity contributions of the separated components at each potential.

A detailed derivation of equations (12 and 13) is given in Supplementary Information. In the following, we proceed to validate the applicability of equation (13), where the average potential ${U}_{-}^{Average}$ is calculated from the linear combination of potential of the constituents. With this purpose, Fig. 4 compares the potential-capacity relationship for a composite electrode with the curves of its separated constituents and the estimation of the composite material assuming a straightforward additivity. With this purpose, the calculated composite curve is obtained adding up the capacities of the separated components at each potential. The composite electrode is made of 25 wt.-% silicon-alloy, 63 wt.-% graphite and 12 wt.-% binder. Details about the materials can be found in the Supplementary Information.

The agreement between the experimental composite capacity and prediction assuming additivity using equation (2) is very satisfactory. Thus, the measurements shown in Fig. 4 support the validity of the model.

The estimation of the total gravimetric capacity of the composite material using equation (2) yields 604 *mAh/g*. This result is obtained adding the contribution (*w*_*j*_ · *s*_*j*_) of each component, 199 *mAh/g* from graphite (black curve) and 404 *mAh/g* from silicon alloy (blue curve) at full lithiation (lowest potential). This estimation results in very good agreement with the experimental value (601 *mAh/g*), with an error below 0.5%. In Fig. 4, this can be appreciated by the coincidence of the red and the dashed green curve at the maximum capacity value.

The experimental average potential of the composite material, 0.384 V, also results in very good agreement with the estimation from the components, which yields 0.385 V using equation (13). The resulting error is below 0.3%.

The example addressed in Fig. 4 supports the assumptions made in the analytical equations for the estimation of the composed anode properties as a function of the basic properties of its components. This type of estimation may be very helpful to accelerate composite electrode design from a practical point of view, since suitable material combinations can be found before electrode fabrication.

For a general material in case study 1, using equations (5, 12 and 13) the volumetric energy density can be calculated as:

14 $$E{D}_{Vol}=({U}_{+}^{Average}-\frac{{\sum }_{j}{U}_{j}^{Average}\cdot {w}_{j}{s}_{j}}{{\sum }_{j}{w}_{j}{s}_{j}})\cdot \frac{{\sum }_{j}{w}_{j}{s}_{j}}{{\sum }_{j}\frac{{w}_{j}}{{\rho }_{j}}+{\sum }_{j}\frac{{w}_{j}}{{\rho }_{j}}\frac{{e}_{j}}{100}}$$

**Figure 5:**
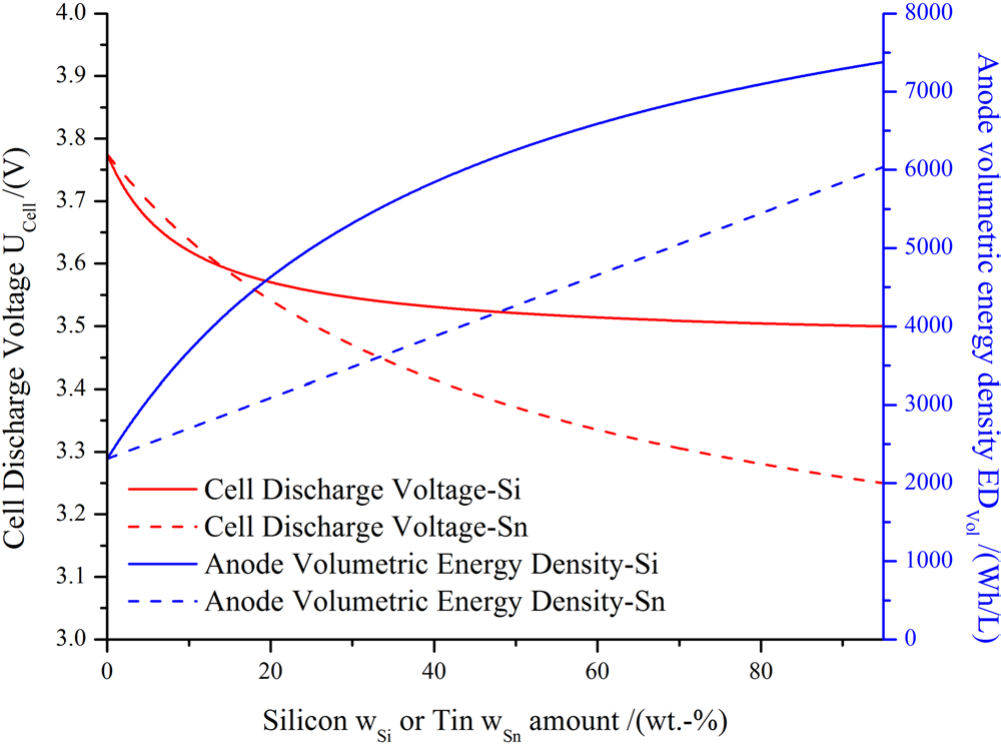
Anode volumetric energy density *ED*_*Vol*_ (in Wh/L) and average cell potential *U*_*cell*_ (in V) obtained according to equations (13 and 14) for Si/Graphite and Sn/Graphite anodes are shown in case-study 1 and LCO cathode. The average potential considered for Graphite, Silicon, Tin and LCO were 0.125 V, 0.400 V, 0.650 V and 3.900 V respectively^3^.

The resulting curves for *ED*_*Vol*_ and *U*_*cell*_ obtained using equations (13 and 14) for Si and Sn are shown in Fig. 5. This Figure shows that the loss of potential difference of the cell by increasing the amount of the active metal is compensated by the increase of volumetric capacity of the material, leading to a total increase of the *ED*_*Vol*_ for the maximum amount of metal material used in the present case (95 wt.-%). Of course this does not take practical cycling stability and mass-transport limitations into account which will be a deciding factor for applications.

The presented case-studies are based on assumptions of theoretical boundary examples regarding the expansion behavior of the materials and the whole composite structure. An electrode reaching zero porosity is not feasible since it will have huge effects on mass transport and rate performance that have not been considered here. These limitations have been investigated by Chandrasekaran *et al*. by explicitly investigating a model based on porous electrode theory^18–20^ which takes into account the varying porosity and the resulting effects on mass-transport. They also assume a case where zero porosity will be reached. Nevertheless their equations were applied to an electrode consisting of one particular active material only and needed the solution of several differential equations for mass-balance and transport equations^13,14^. Instead, the examples here presented show how the derived equations are easily applied to aid in the design and comparison of relevant electrode structures. Since the developed equations are applicable to arbitrary materials, porosities and expansion tolerance factors; a detailed experimental investigation for each material combination is of practical importance. We only focused on cases where the active materials reach full lithiation. The derived model equations can also be used to investigate cases with restrictions to the state of charge, which are commonly applied in commercial batteries to extend lifetime. A detailed analysis of these cases should take into account that different active materials will have different lithiation rates, though. Introducing stress-effects might add another important variable for the practical design of composite electrodes. Although more experimental as well as theoretical research needs to be done on this topic, we include an additional calculation in the Supplementary Information that takes into account the effect of mechanical stress on silicon lithiation potential and anode volumetric energy density. According to theoretical and experimental work done by Sethuraman *et al*.^21^, the application of a stress Δ*σ* to silicon thin films changes the potential by the amount *γ*Δ*σ* for any state of charge. Experiments show that *γ* = 110 *mV*/*GPa*, indicating that a compressive stress (Δ*σ* < 0) will decrease the silicon lithiation potential, increasing the volumetric energy density. The opposite will happen for tensile stress (Δ*σ* > 0). If, for example, we study the case of a compressive stress of Δ*σ* = −1 *GPa* acting on the electrode (either through the expansion of the particles or due to a mechanical constriction via the cell casing) the increase on the anode volumetric energy density at maximum silicon content (95 wt.-%) will be of 3%. This small increase in the volumetric energy density must be contrasted with the fact pointed out by Wang *et al*.^22^ that the compressive stress decreases the silicon ionic conductivity (lithium ion diffusion). Similar analysis could be done for the particular case of silicon nanowires taking into account the results of Zhang *et al*.^23^. We are aware of the fact that we neglected possible stress effects on graphite^24^ in this approximation, but just wanted to emphasize the flexibility of the equations put forward.

### Searching for a universal kinetic behavior of silicon electrodes

**Figure 6:**
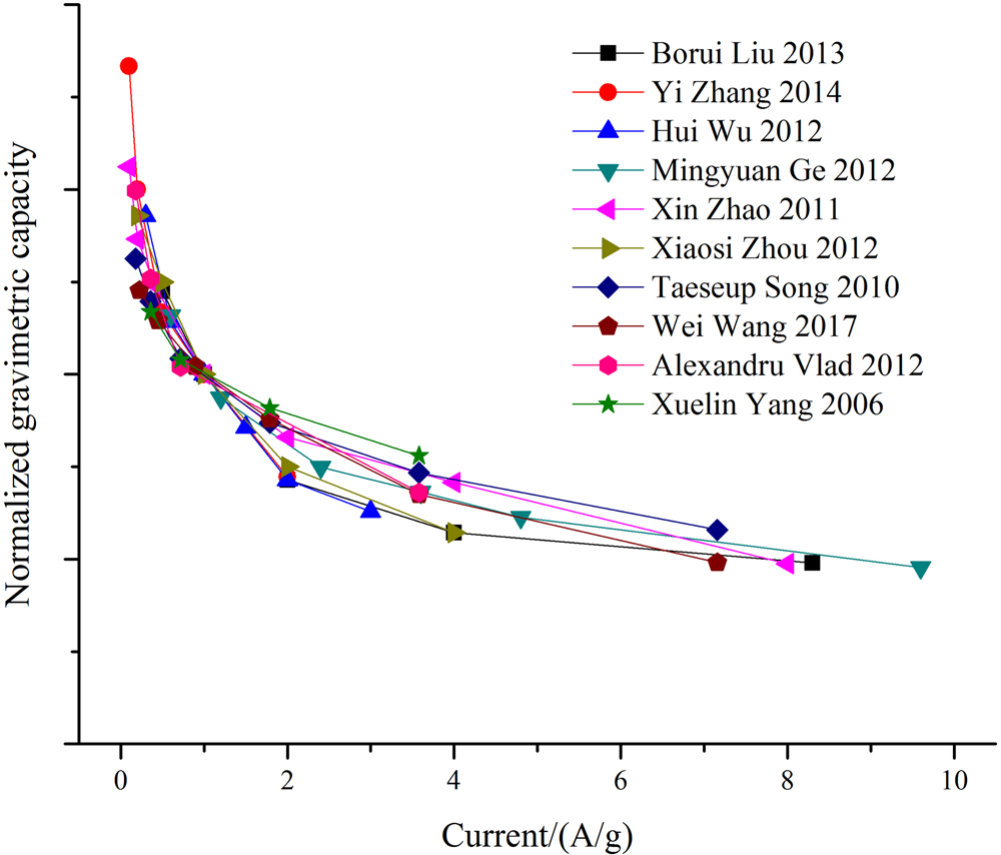
Normalized gravimetric capacity as a function of the current density for a wide variety of electrodes composed of nanostructured silicon materials^29–38^. For detailed discussion see Supplementary Information.

Silicon based electrodes present a decreasing gravimetric capacity for increasing current densities (see Supplementary Fig. S5). This behavior is due to the kinetic properties of the lithiation/delithiation process of silicon^25^, since this is the limiting step in electrode charging and discharging. Although the accessible capacity greatly depends on the material preparation, it is heuristically found (see Supplementary Information) that the behavior of a wide variety of electrodes composed of nanostructured silicon materials can be cast into a general picture. Normalized gravimetric capacity curves follow the same comportment, as can be noticed in Fig. 6.

**Figure 7:**
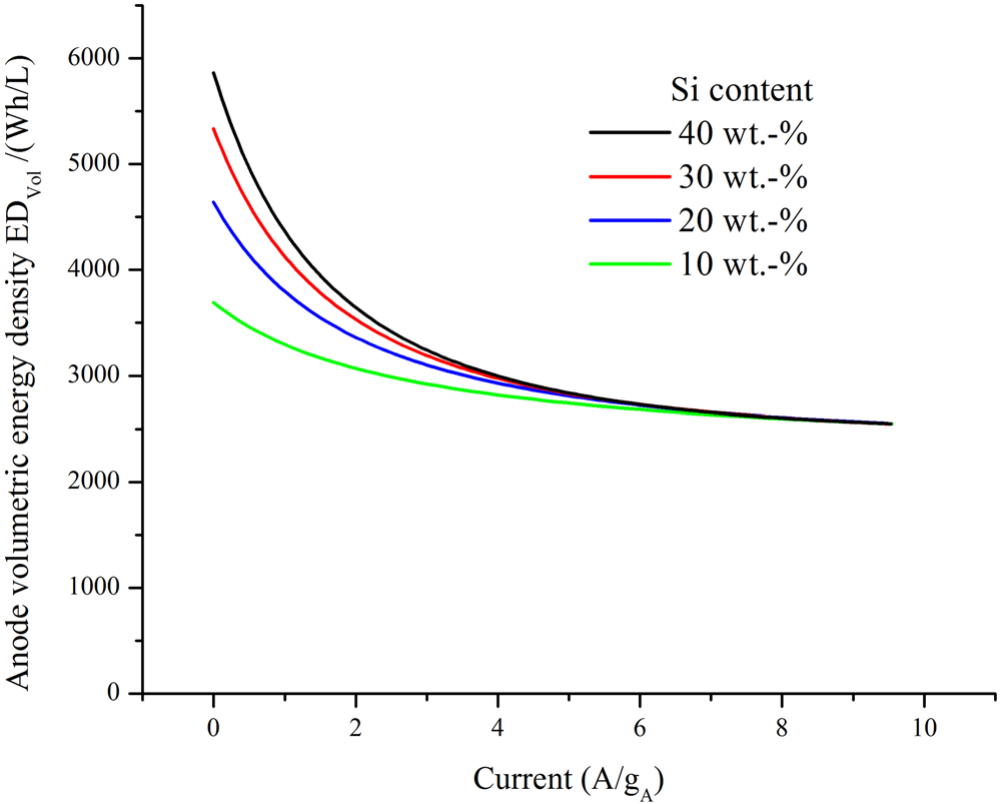
Anode volumetric energy density estimations as a function of delithiation anode current density for silicon/graphite composite electrodes of different compositions in case-study 1 versus a LCO cathode. The average potential considered for Graphite, Silicon, and LCO were 0.125 V, 0.400 V, and 3.900 V respectively.

Several attempts have been made to find a general empirical rule to analyze capacity loss upon increased discharging rate in numerous electrode materials^26,27^. Figure 6 shows a collapse of several experimental results that allows finding a general functional relation to estimate (or fit) experimental rate capability data. This empirical relationship, in combination with equation (14), can be used to calculate the variation of the achievable electrode volumetric energy density *ED*_*Vol*_ as a function of the lithiation/delithiation current density, as shown in Fig. 7. A detailed explanation of the mathematical steps undertaken with this goal can be found in Supplementary Information.

A number of interesting conclusions can be drawn from the analysis of Fig. 7. The functional form of the decrease in *ED*_*Vol*_ for increasing current density resembles the loss in gravimetric capacity shown in Fig. 6. The loss in *ED*_*Vol*_ is pronounced for currents up to 2 A/g_A_ and less significant for higher values. The performance decrease is more prominent for samples with higher silicon content, leading to a similar value for high current densities. Taking this result into account, it can be concluded that if a high current density anode is pursued, high silicon content will not be reflected in a higher *ED*_*Vol*_. Another effect of the increase in current density will be an increasing overpotential^13,28^, leading to a decrease of the cell potential and a further decrease in *ED*_*Vol*_. This effect is less pronounced than the previous one and does not change the main features of the functional form of Fig. 7. A detailed analysis of this topic and its implications is developed in Supplementary Information.

## Conclusions

We have developed a set of analytical equations suited to undertake design considerations for combined active materials in practical lithium ion-battery electrodes. The equations can be applied to any material combination for positive or negative electrodes as long as basic parameters are available. Silicon-graphite and tin-graphite composite electrodes were chosen as relevant examples. Our calculations show that the increase of the amount of metal active material will always increase the volumetric and specific capacity of the electrode. Interestingly, we find that the functional behavior of the volumetric capacity in regard to increasing metal active material content is very different for the considered examples.

In the case of silicon-graphite electrode the volumetric capacity shows a rapid increase for low Si content, which slows down for higher Si concentrations. Thus, comparatively big gains can only be achieved with silicon contents up to 40 wt.-%. Beyond this point the rate of increase becomes almost constant and gains in energy density with increasing silicon amount become less pronounced. The present results show that choosing silicon over tin is always the best alternative, but less than expected if one considers structural stability. Average delithiation potentials were estimated for a composite electrode and contrasted with experimental measurements, showing that the used analytical equations correctly describe the electrode behavior.

Finally, on the basis of experimental data from the literature, a very general empirical relationship describing the dependence of the capacity of the material on the delithiation current for silicon nanostructured electrodes was found. This empirical relationship was used to illustrate how the analytical equations can be used to study the influence of kinetic limitations on the capacity of the material.

## Method

To perform the described calculations we utilized common spreadsheet-software products. The equations were derived by assuming boundary constrictions for the electrode structure and solving them based on common manufacturing quantities like weight fractions and electrode porosity. For the energy density calculations we assumed a LiCoO_2_ cathode with an average potential of 3,9 V over the whole state of charge.

The electrodes were prepared by weighing and then mixing the active materials in an aqueous solution of polyacrylic acid (PAA, Mw ~250.000, 35 wt.-% in water, Sigma Aldrich). The mixing was conducted in a 80 ml stainless steel milling vial with ten 10 mm diameter stainless steel balls at a revolution speed of 100 rpm for 30 minutes in a Retsch PM 400 MA ball mill. Deionized water was added to the suspension until a suitable viscosity for coating was achieved. The coating was performed by the doctor-blade method with a coating gap height of 100 µm. A 20 µm thick copper foil was used as substrate and current collector. The coating was left to dry under air for 24 h and successively dried under vacuum at 80 °C before being taken into an argon-filled glovebox. Electrodes of 20 mm diameter were punched and assembled in Swagelok-type half-cells versus lithium-metal electrodes. 15 µl of electrolyte were added. The electrolyte was 1 M LiPF_6_ in EC/DEC/FEC in relative volumetric amounts of 3/6/1. The electrodes were cycled with a three steps procedure. First, a constant charging current was applied until reaching the lower cut-off potential of 0.01 V versus Li/Li^+^. Then the potential was held constant at this lower cut-off potential until the current declined to a value of C/20. Lastly, a constant-current discharge was performed. The current during galvanostatic charging and discharging was respectively C/10 for the first cycle and C/4 for the following cycles.

## Electronic supplementary material

Supplementary Information
