# Supplementary material for: Design-Considerations regarding Silicon/Graphite and Tin/Graphite Composite Electrodes for Lithium-Ion Batteries
Source: Sci Rep. 2018 Oct 26;8:15851. doi: 10.1038/s41598-018-33405-y (PMC6203704; doi:10.1038/s41598-018-33405-y)
Supplement: Supplementary file 1 — Supplementary Information [file 41598_2018_33405_MOESM1_ESM.doc]

**Supplementary Information for:**

**Design-Considerations regarding Silicon/Graphite and Tin/Graphite Composite Electrodes for Lithium-Ion Batteries**

**Manuel Otero ^1,2^, Christopher Heim ^3^, Ezequiel P.M. Leiva ^2^, Norbert Wagner ^3^, Andreas Friedrich ^3,4^**

^1^ IFEG, Facultad de Matemáticas, Astronomía y Física, Universidad Nacional de Córdoba, Córdoba, Argentina

^2^ INFICQ, Departamento de Química Teórica y Computacional, Facultad de Ciencias Químicas, Universidad Nacional de Córdoba, Córdoba, Argentina

^3^ German Aerospace Center (DLR), Institute of Engineering Thermodynamics, Pfaffenwaldring 38-40, 70569 Stuttgart, Germany

^4^ Institute for Energy Storage, University of Stuttgart, Pfaffenwaldring 31, 70569 Stuttgart, Germany

**Derivation of equation (8) and comparison with equation (3) of Dash *et al.*^8^**

Starting from equation (6):

(S1)

(S2)

(S3)

Dividing equation (S3) by $V^{i}$ yields:

(S4)

Defining the initial volumetric percentage of each material as leads to:

(S5)

From which equation (7) may be obtained

(S6)

which is the relationship sought between the initial and final porosities. Obviously, it depends on the initial and final volumes, if the latter is allowed to change. On the other hand, if the volume is fixed ($V^{f}=V^{i}$) as in case-study 1, the final porosity will be given by equation (8):

(S7)

Thus, considering ($V^{f}=V^{i}$) and using a silicon/graphite composite material whose expansions are $e_{\text{Si}}=280$vol.-% and $e_{G}=10$vol.-% respectively, equation (S7) yields:

(S8)

The previous equation is the correct relation between the initial and the final porosity.

Although this equation is similar equation (3) from reference [8], it has a fundamental difference. ***Instead of depending from the weight percentages $w_{\text{Si}}$ and $w_{G}$ , this equation depends on the volumetric percentages $v_{\text{Si}}$ and $v_{G}$ .***

In the following paragraph we analyze if equation (S8) may lead to equation (3) from reference [8] under certain conditions. In order to do this, equation (S8) may be written in terms of weight fractions and densities:

Where $m_{j}$is the mass of each component *j*. Defining the initial weight fraction $w_{j}$ (in wt.-%) of each material as a function of the total mass of the electrode M:

Then the volumetric fraction of each component $v_{j}$ can be obtained by:

(S9)

Considering that $\rho_{A}=\frac{M}{V^{i}}$ is the initial density of the electrode, yields:

(S10)

Inserting this in equation (S8), leads to:

(S11)

Since the initial density of silicon and graphite considered in reference [8] are similar, and , it could be assumed that to get:

(S12)

For an electrode composed **only** of silicon and graphite (without any inactive materials), assuming that they have the same density and **neglecting the initial porosity**, it can be stated that the density of the electrode is equal to that of one of the components $\rho_{A}=\bar{\rho}$ and **then equation (3) from reference [8]** is obtained:

(S13)

But this equation is **only** an approximation valid for electrodes composed entirely of materials of the same density (which is an acceptable approximation for silicon and graphite) and with **no initial porosity**. Reference [8] uses this equation for electrodes with initial porosities between ~23% and ~95%, something which leaves to incorrect results and conclusions.

Alternative derivation of equation (8):

Since for Case-Study 1 the volume increase of the active materials decreases the available porosity and the total volume is fixed ($V_{i}=V_{f}=V$), the initial and final porosities can be defined as:

Then the change in the available porosity will be:

Using the definition of initial volumetric fraction, $v_{j}$, equation (8) is obtained:

**Influence of the amount of inactive material in Case-Study 1**

Calculations for Silicon/Graphite composites in case-study 1(similar to those reported in Figure 2) were performed for different inactive material compositions, and are shown in the following Figure:


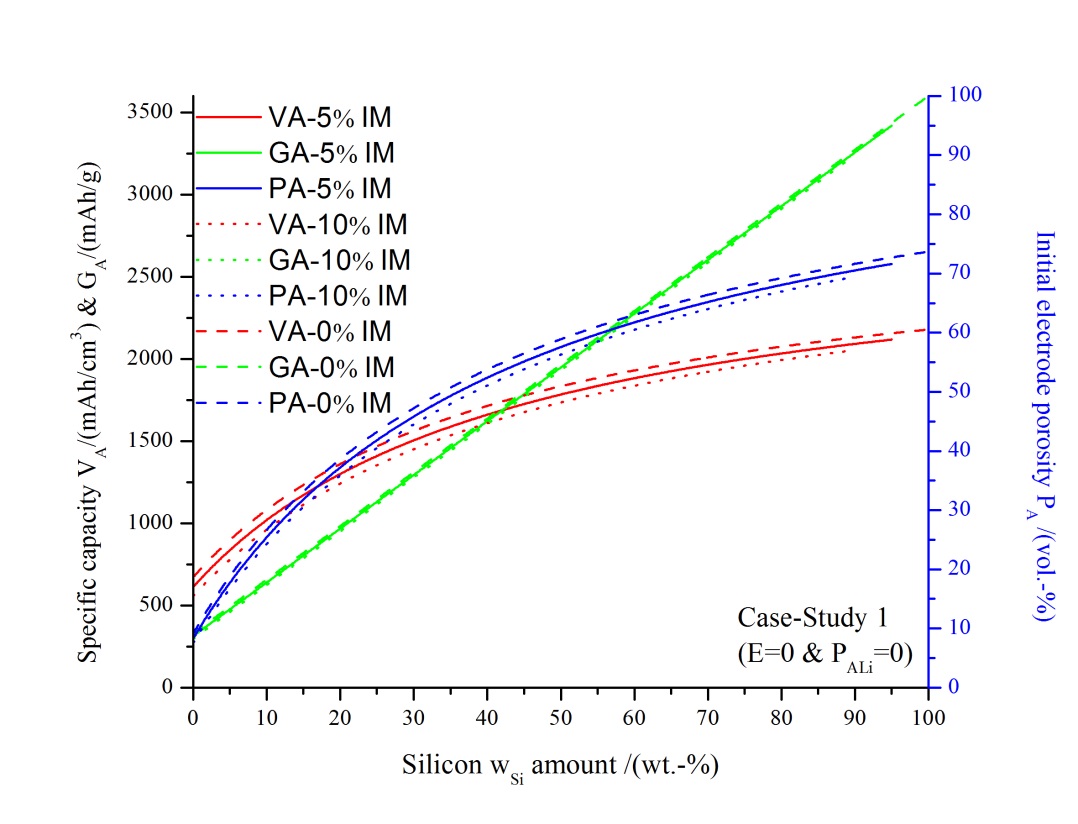


Supplementary Figure S1: Silicon/Graphite composites in case-study 1(same parameters as in Figure 2) for different amounts of inactive materials: 0 wt.-% (points), 5 wt.-% (full line) and 10 wt.-% (dashed line).

The previous figure shows the effect of using different amounts of inactive material on the specific capacity and initial electrode porosity for a Silicon/Graphite composite anode in case-study 1. We compare the 5 wt.-% used in Figure 2 of the present manuscript with 10 wt.-% and without (0 wt.-%) the use of inactive material. Although the use of higher amounts of inactive material will decrease the capacity of the anode, even commercial graphite electrodes have a binder and carbon black content of around 2 wt.-% combined. It is expected that materials such as silicon and tin will need higher amounts of inactive materials to overcome the lower electronic conductivity and significant structural changes.

It can be inferred from the previous figure that the qualitative behavior of the anode properties as a function of the composition does not change appreciably with the content of inactive material. The readers may use the equations derived in the body of the article to study different case-studies and materials of their own interest.

**Case-study 2 for Tin/Graphite negative electrodes**


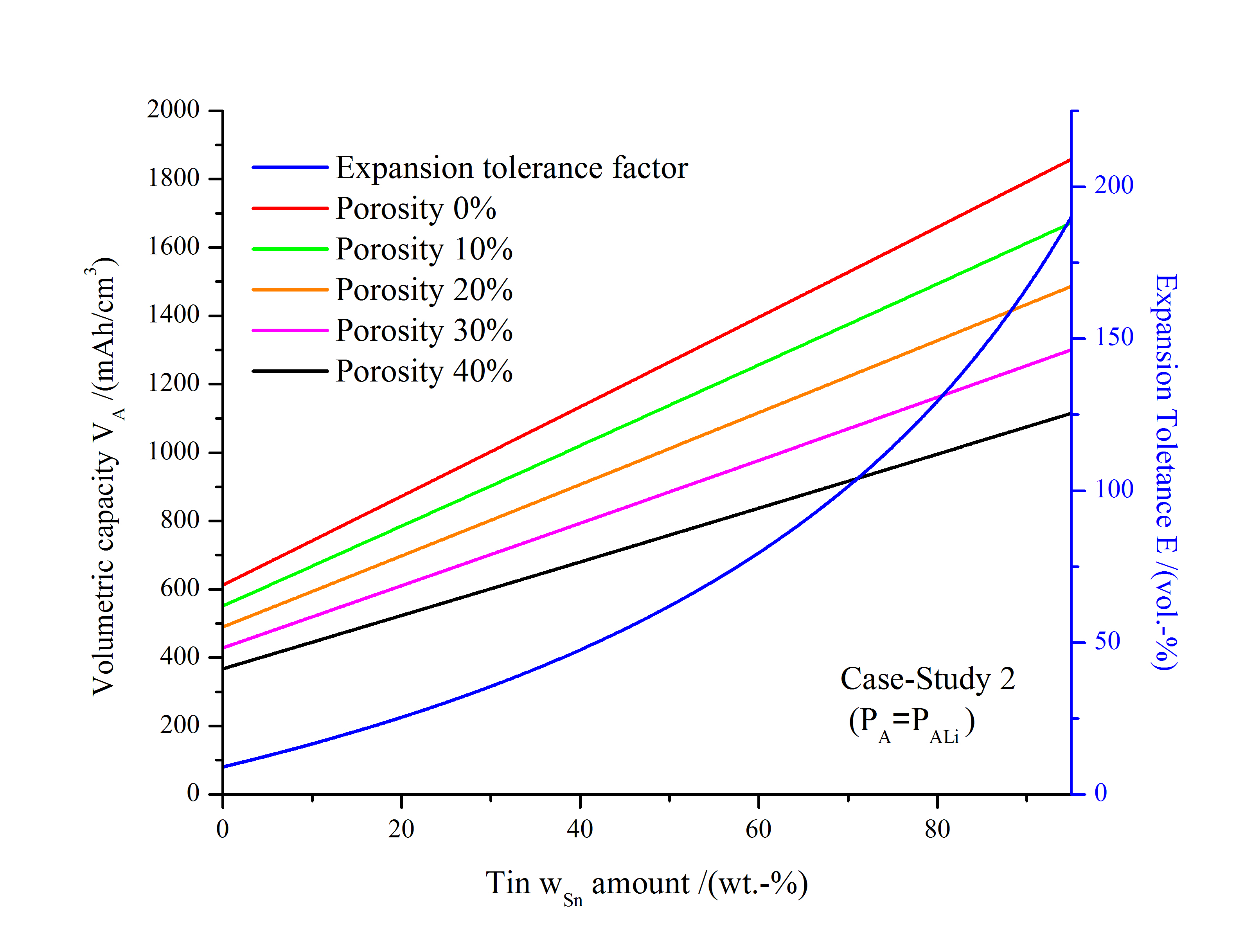


Supplementary Figure S2: Volumetric capacities ($V_{A}$ in mAh/cm^3^) of tin/graphite composite electrodes for different porosities (0, 10, 20, 30 and 40 vol.-%) under the condition of a constant porosity are shown. The expansion tolerance E (in vol.-%) required to keep a constant porosity upon lithiation is drawn with the blue line. The parameters considered were, $P_{\text{ALi}}=P_{A}$*,*, , ^6^, , , $e_{\text{Sn}}=275$vol.-% ,$e_{G}=10$vol.-%, $w_{\text{IM}}=5$wt.-%.

**Derivation of average anode potential**

Obrovac et al. [7] have given an equation to obtain the volumetric energy density of an anode material in a lithium-ion cell from the voltage of the anode and cathode
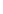
of the cell, say and . In the previous work, was calculated from the integration of the cell potential difference as a function of the number of moles of lithium per mole of host alloy atoms, , according to:

(S14)

The previous equation is given as equation (3) of the article of Obrovac [7], where is the number of moles of lithium per mole of host alloy atoms at full lithiation, is the molar volume of the alloy anode (calculated per mole of host alloy atoms), and F is Faraday’s number in units of Ah/mol (). Thus

(S15)

(S16)

For the simplest case where the potential for the negative and positive electrode are constant upon discharging, and we have:

(S17)

Where using equation (S15) and (S16) leads to:

(S18)

And since :

(S19)

(See equation (3) of the present article). Then equation (S17) results:

(S20)

This is equation (12) in the present article or equation (13) from Obrovac et al. [3]. In a more realistic case where the anode potential $U_{-}(x)$ changes upon discharging but taking the cathode potential to be constant (which is a good approximation), we have:

(S21)

Which, using equation (S19), results in:

(S22)

Using the definition of the average of a function in a given range $x\in[a,b]$:

It can be noticed that the last term of equation (S22) is the definition of the *average anode potential* upon discharging

(S23)

So that the anode volumetric energy density is:

(S24)

One can take any independent variable to perform the integration if it covers the whole range of interest. For an easier correlation to our description we will take the gravimetric capacity instead of the number of moles of lithium per mole of host alloy atoms used by Obrovac et al. [7]. Then equation (S23) becomes:

(S25)

The previous equation can be formulated, since there is a one to one relationship between and . In equation (S25) is the anode gravimetric capacity as defined in equation (2) and is the partial amount of charge (lithium atoms) calculated per weight of host alloy atoms upon discharge. Obrovac et al. [7] developed the concept of average voltage for the study of delithiation of a Si electrode, illustrated in Figure 3 of their manuscript. A similar plot is given below in Supplementary Figure S3 (left plot) for clarity. At this point it is interesting to notice that the value of will not change if one uses the *average anode potential* as in equation (S24) or the anode potential function as in equation (S22). This is illustrated in Figure 4 of Obrovac’s work [7] considering the cell at fully discharged state.

In this approach, the sloping voltage of the experimental curve is substituted by a mathematical step (Heaviside function). We illustrate on the left plot below the approximation made by these authors and on the right the extension for a system where the discharge is produced in an electrode which has two active components. Experiments showing this behavior can be found in the literature [15, 16, 17]. Also Figure 4 of the present manuscript presents experimental measurements of a composite negative electrode to validate this approach.

| 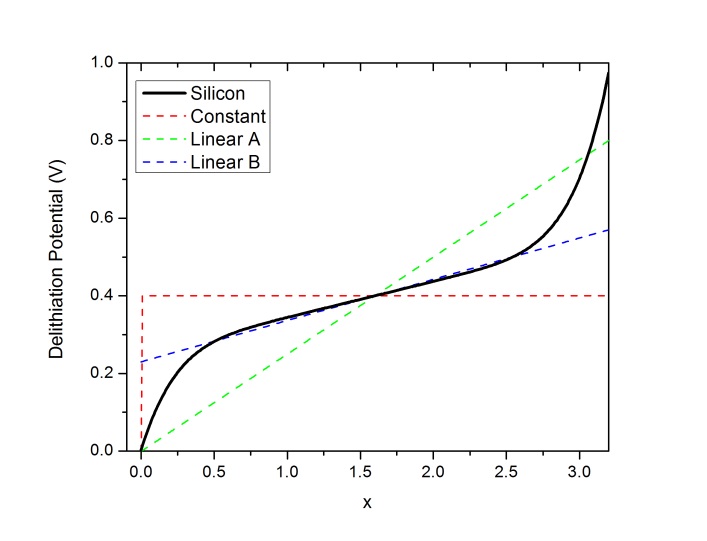 | 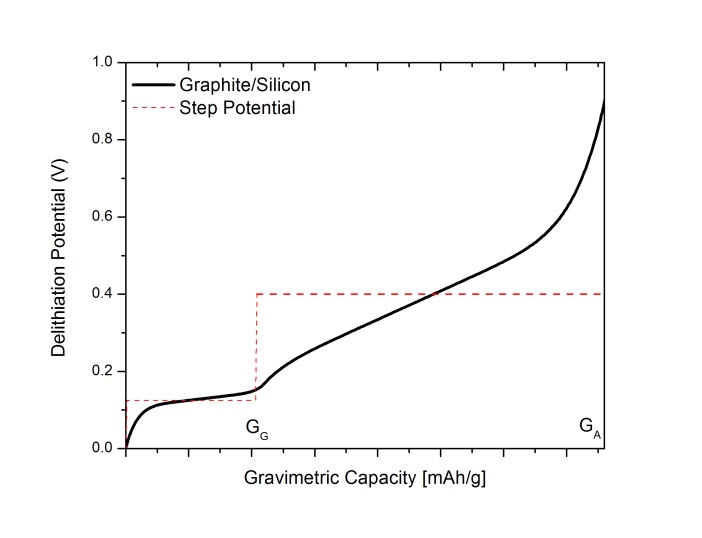 |
| --- | --- |

Supplementary Figure S3: Schematic discharge voltage profile of a silicon anode (left side) and a graphite/silicon composed anode (right side). Full line represents the complex experimental curve and dashed lines are different representations leading to the same energy density at full discharge. The average voltage is the same for all voltage curves.

On the right, we show our approximation for the discharge of the two materials constituting the electrode that results in the occurrence of two steps. There, each of the two sloping sections corresponding to the discharge of each material is replaced by an average potential value. In other words, we assume that the discharge curves of the two materials show little overlap, so that each of the processes (discharge of the Si electrode and discharge of the carbonaceous material) may be replaced by a step.

The function representing the average potential is:

(S26)

Where represents the average discharge potential of each material. Since the delithiation potential of the carbonaceous material () is below the delithiation potential of silicon (), we assume a complete delithiation of the carbonaceous material before silicon participates. The average potential of the composite anode will depend on the capacity of the carbonaceous material and the silicon . The calculation of the average potential using (S25) will be:

(S27)

(S28)

With

(S29)

So we finally get:

(S30)

In a general case for an anode made of properly separated j components we get equation (13). The previous approach is very close to that used by Ng et al. in reference [15].

**Details about material preparation**

To stabilize the volume expansion of silicon for practical application in the composite electrode, a silicon alloy was used. The silicon alloy active material was prepared by high energy mechanical milling. Appropriate amounts of elemental Si (-325 mesh), elemental Fe powder (50nm) and fumed silica (~50 nm) were milled for 40 h inside an 80 cm³ stainless steel milling vial with 25 stainless steel balls of 10mm diameter. The vial was closed under argon atmosphere and the milling process was conducted on a Retsch PM 400 MA at 400 rpm. The resulting particles had a size of approximately 5 µm.

**Effect of stress on the volumetric energy density**

According to Sethuraman et al. ([21], equation (15)) stress affects the silicon lithiation potential as follows:

(S31)

Where $\Delta\sigma$ is positive for a tensile stress and negative for a compressive stress. The parameter $\gamma$ was theoretically calculated to be and the experimental value resulted to be around . Since this potential variation was found to be constant for different states of charge, this can be added to our calculation as:

(S32)

Using equation (S25) to calculate the average potential of silicon and taking into account stress, yields:

(S33)

Using the previous definition of for the case of zero stress we get:

(S34)

Thus, the anode average potential upon stress results:

(S35)

And the anode volumetric energy density (S24) is:

(S36)

From equation (S35) and (S36) it can be noticed that a compressive stress ($\Delta\sigma<0$) will lead to a decrease in the potential of the anode, to an increase in the cell potential and to an increase in the anode volumetric energy density. As an example, we show in Supplementary Figure S4 the effect of a compressive stress assuming .


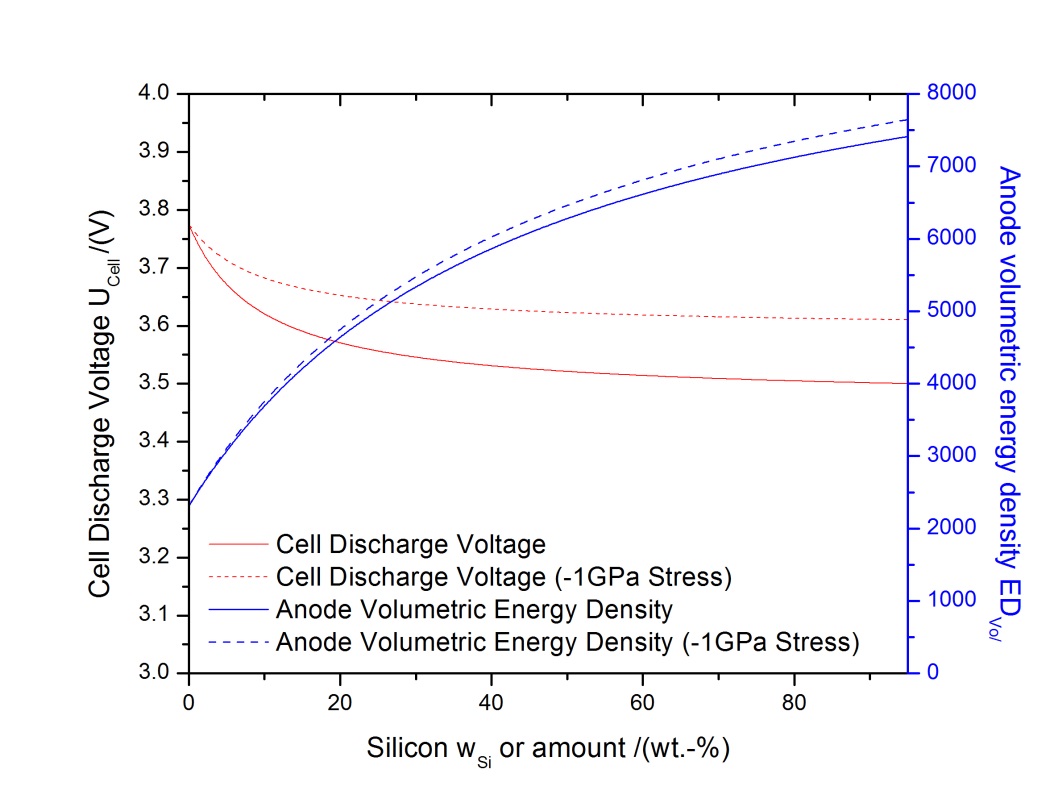


Supplementary Figure S4: Cell discharge voltage ($U_{\text{Cell}}$ in V) and anode volumetric energy density ( in Wh/L) of Si/graphite composite electrodes for cero stress (full line) and for (dashed line) are shown for case-study 1 and a LCO cathode. The average unstressed potentials considered for Graphite, Silicon and LCO were 0.125 V, 0.400 V and 3.9 V respectively.

Assuming a constant stress over the whole discharging step is certainly a simplification, but the goal here is to show that several relevant physical phenomena can be added to the equations to improve their relevance. The extension to variable stress effects would require replacing the constant stress value by a suitable function, which would depend on the state of charge and/or temperature, but the general implementation would be straight-forward as shown above. Additionally we would like to mention here that we only demonstrated the extension of the equations to take into account stress-effects on silicon.

**Searching for an universal kinetic behavior of silicon electrode**

The following figure shows the gravimetric capacity as a function of the current density of several nanostructured silicon electrodes [26-35]. This kinetic behavior presents a similar trend for all the data as can be noted in Supplementary Figure S5.


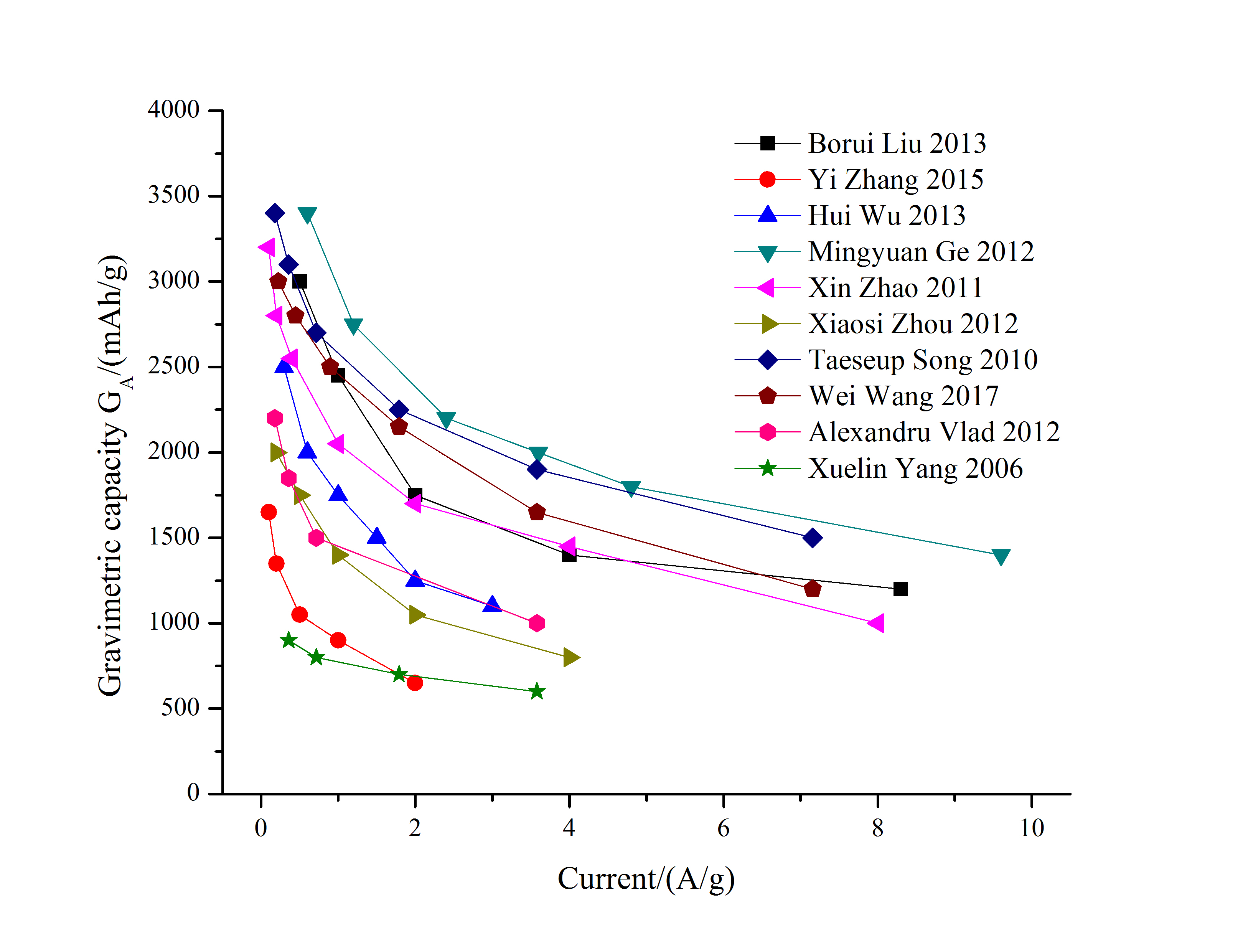


Supplementary Figure S5: Gravimetric capacity as a function of the current density for a wide variety of electrodes composed of nanostructured silicon materials. Date taken from references [26-35]

To allow the comparison between the results of different composite materials, the absolute value of the gravimetric capacity was normalized using the following relation:

(S37)

Where represent the gravimetric capacity at current i. This normalization allows a comparison of the functional form of the gravimetric capacity as a function of the current density for different electrodes.

Several functional forms where tested to fit these measurements, see Supplementary Figure S6 and Supplementary Table S1.


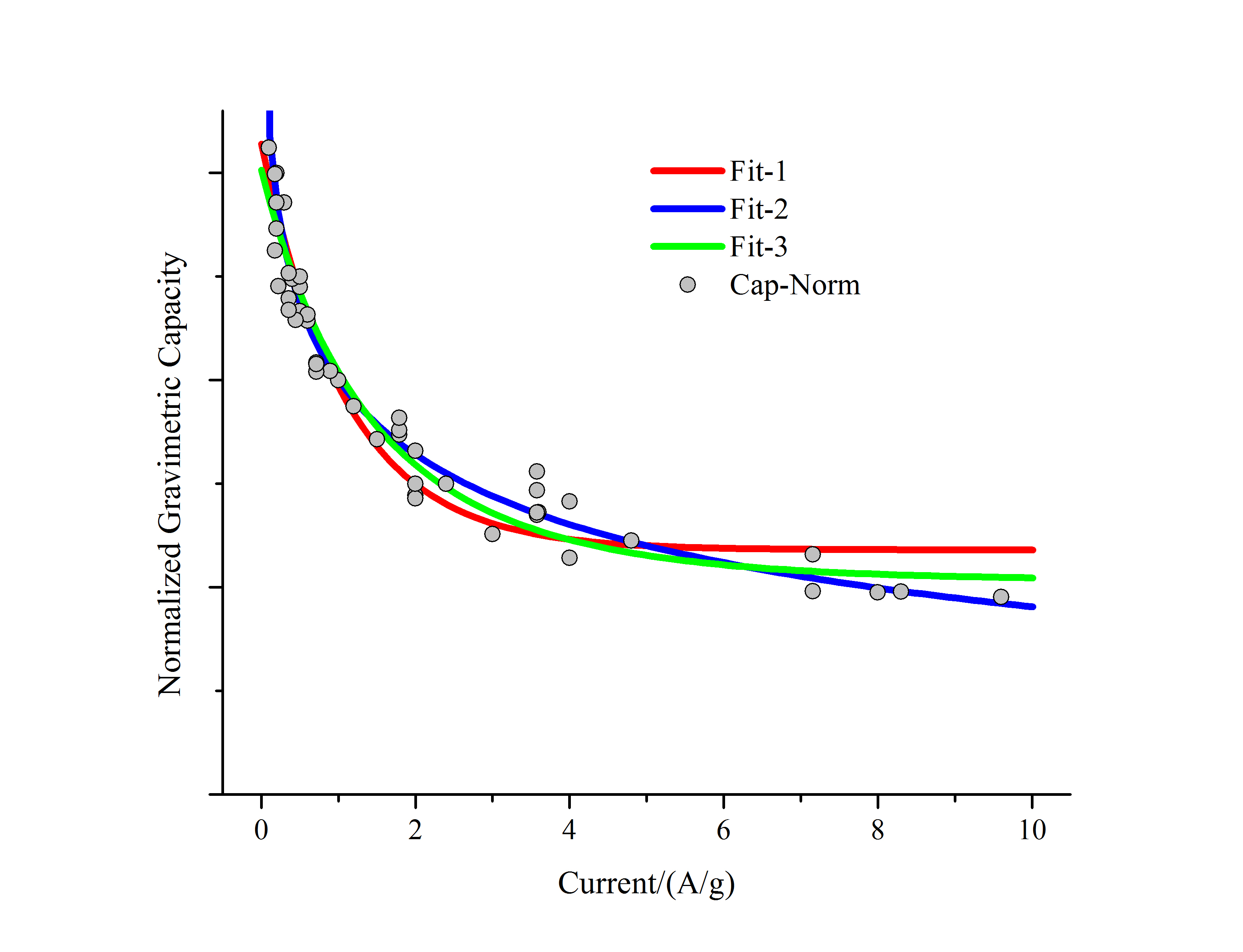


Supplementary Figure S6: Normalized gravimetric capacity fit by 3 different functions.

| Fit Number | Functional Form | Fitted parameters |
| --- | --- | --- |
| 1 |  |  |
| 2 |  |  |
| 3 |  |  |

Supplementary Table S1: Fitting functions in Supplementary Figure S6.

Using any of the fitted functions in equation (14), $\text{ED}_{\text{Vol}}$ can be obtained as a function of the current density for different anodes containing silicon. Figure 7 presents the results for fitting function 3. This figure provides a useful tool to predict the kinetic behavior of different Si-contaning anodes.

Another effect of the increase of the current density used to discharge the electrodes is that an increasing overpotential. Which leads to a decrease of the cell voltage and thus to a further decrease in $\text{ED}_{\text{Vol}}$. This fact has been illustrated in the experimental work of Tushar Swamy et al. [38] on silicon particles. Three important conclusions may be drawn from the results presented in Figure 4 of reference [38]:

- The increase of silicon overpotential for increasing C-rate (discharge current) follows a square root behavior.
- The overpotential also depends on particle size (surface/volume relation), but the functional form remains unchanged.
- For graphite the effect on the overpotential is one order of magnitude smaller than in the case of Si.

Also the theoretical work of Chandrasekaran et al. [13] predicts a square root functional behavior for the overpotential as a function of current density in silicon nanoparticles.

Thus, it comes out that the discharge overpotential found for electrodes made of Si nanoparticles can be fitted by a simple square root function to predict its behavior as a function of the discharge current. This functional fitting can be used to correct the average potential in equation (13), leading to a correction in the $\text{ED}_{\text{Vol}}$ values. These corrections are shown in the following Figure for 40% silicon content composite anodes. It is found that the correction slightly lowers$\text{ED}_{\text{Vol}}$, but the overall functional shape remains unchanged.


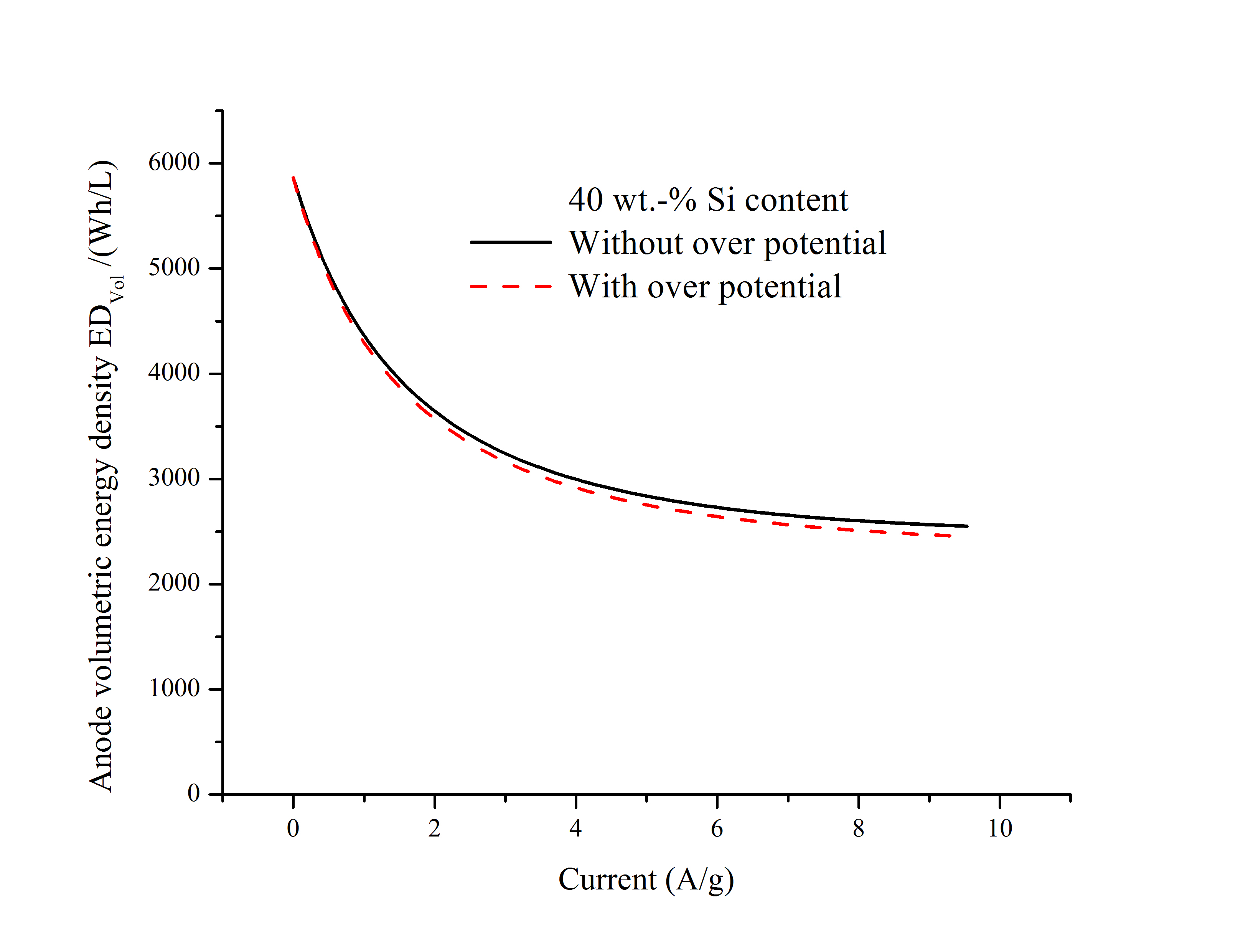


Supplementary Figure S7: Anode volumetric energy density estimations as a function of delithiation anode current density for a silicon/graphite composite electrode of 40 wt-% Si content in case-study 1 versus a LCO cathode. The average potential considered for Graphite, Silicon, and LCO were 0.125 V, 0.400 V, and 3.9 V respectively. The case where overpotential due to high currents is considered (dash red) is compared with the case where overpotential is omitted (black).

The following figure shows $\text{ED}_{\text{Vol}}$ as a function of the current density for electrodes with different Si/Graphite compositions, taking into account the silicon overpotential effect. The overpotential for graphite can be neglected, since it is one order of magnitude smaller.


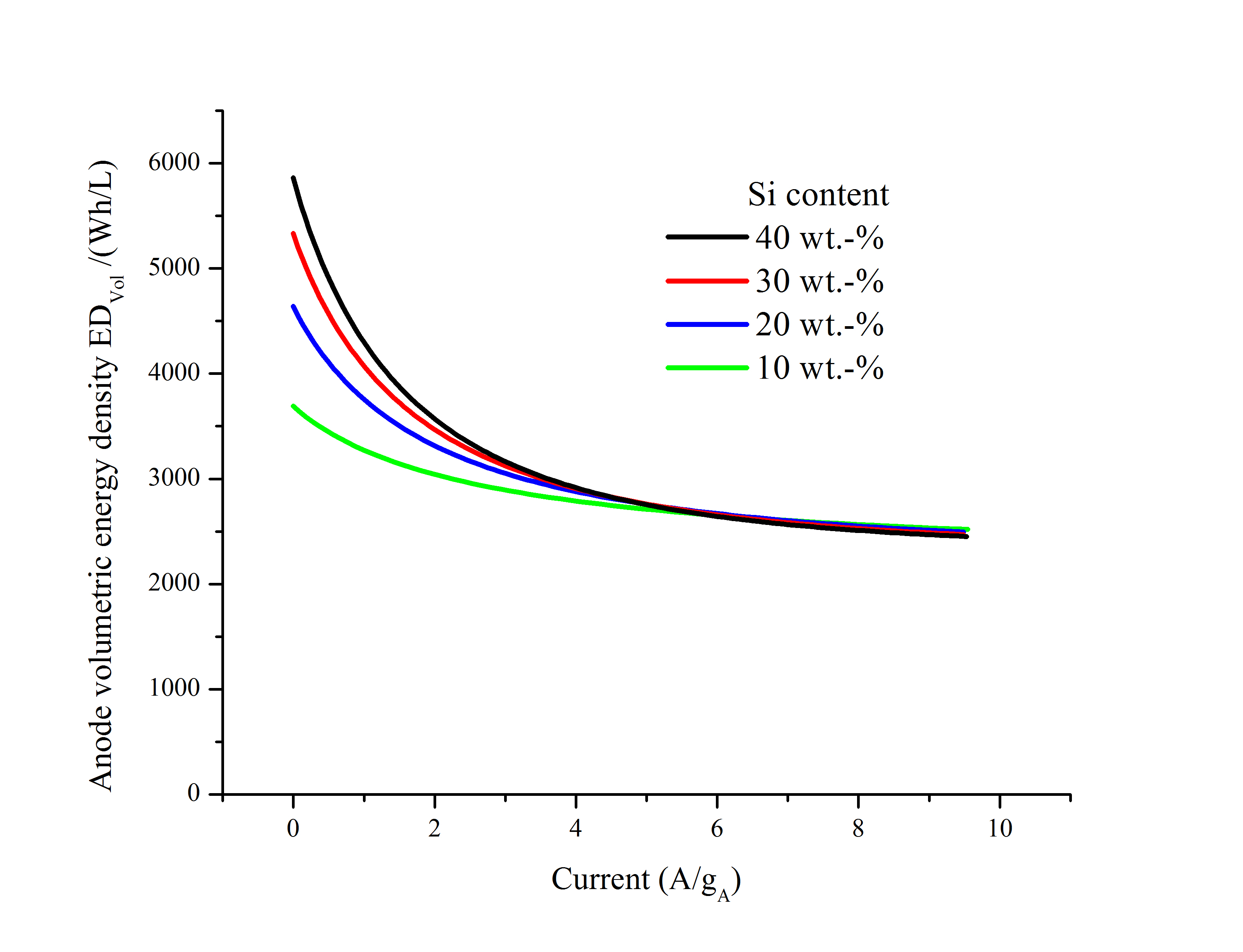


Supplementary Figure S8: Anode volumetric energy density estimations as a function of delithiation anode current density for a silicon/graphite composite electrode of different Si content in case-study 1 versus a LCO cathode considering the overpotential due to high currents. The average potential considered for Graphite, Silicon, and LCO were 0.125 V, 0.400 V, and 3.9 V respectively.

It can be seen that the behavior of $\text{ED}_{\text{Vol}}$ taking into account the silicon overpotential is similar to the one shown in Figure 7, so that all the conclusions remain valid.
